# Mortality, perception, and scale: Understanding how predation shapes space use in a wild prey population

**DOI:** 10.1371/journal.pone.0222272

**Published:** 2019-09-25

**Authors:** Lindsey N. Messinger, Erica F. Stuber, Christopher J. Chizinski, Joseph J. Fontaine

**Affiliations:** 1 Nebraska Cooperative Fish & Wildlife Research Unit, University of Nebraska–Lincoln, Lincoln, Nebraska, United States of America; 2 School of Natural Resources, University of Nebraska–Lincoln, Lincoln, Nebraska, United States of America; US Geological Survey, UNITED STATES

## Abstract

Attempts to assess behavioral responses of prey to predation risk are often confounded by depredation of prey. Moreover, the scale at which the response of prey is assessed has important implications for discovering how predation risk alters prey behavior. Herein, we assessed space use of wild Ring-necked Pheasants (*Phasianus colchicus*) in response to spatial and temporal variation in recreational hunting. We radio-marked pheasants and monitored space use at two spatial scales: short-term seasonal home range, and nightly resting locations. Additionally, we considered temporal variation in predation risk by monitoring space use prior to and during the pheasant hunting season. Although we found no change in nightly resting location, pheasants subjected to predation risk expanded their home range and shifted home range location even when invulnerable to predation. Home range formation was plastic, with home ranges expanding and contracting as risk fluctuated before and during the hunting season. Depredation reduced the measured response within the population, obscuring the potential importance of perceived predation risk in shaping prey communities, particularly when not measured at the appropriate scale. By assessing space use of a wild prey population at multiple scales, considering spatial and temporal variation in predation risk, we show that not only does predation risk affect space use, but that the effects at the population level may be challenging to assess when not measured at the appropriate ecological scale because of the direct effects of differential mortality on the same behaviors.

## Introduction

Space use has profound implications for individuals, populations and communities [1–5] with variation in resource availability, predation, competition, and climate all influencing how individuals select and use appropriate habitats [4,6–13], and the scale(s) at which responses are manifested [11–13]. Predation risk (i.e., the probability of being killed by a predator as a function of predator encounter rate and time spent vulnerable to predator encounter [14]), for example, alters space use [15–17] such that individuals are found disproportionately in areas with limited exposure to predators (reviewed by [14,18]). Although predation risk is often considered either spatially (risky places hypothesis, i.e., [19,20]) or temporally explicit (risky times hypothesis, [14,18]), prey continually assess and respond to predation risk (e.g., risk allocation hypothesis, [21]), making understanding how predators shape the distribution of prey communities dependent on understanding both spatial and temporal variation in risk [22–25].

**Fig 1:**
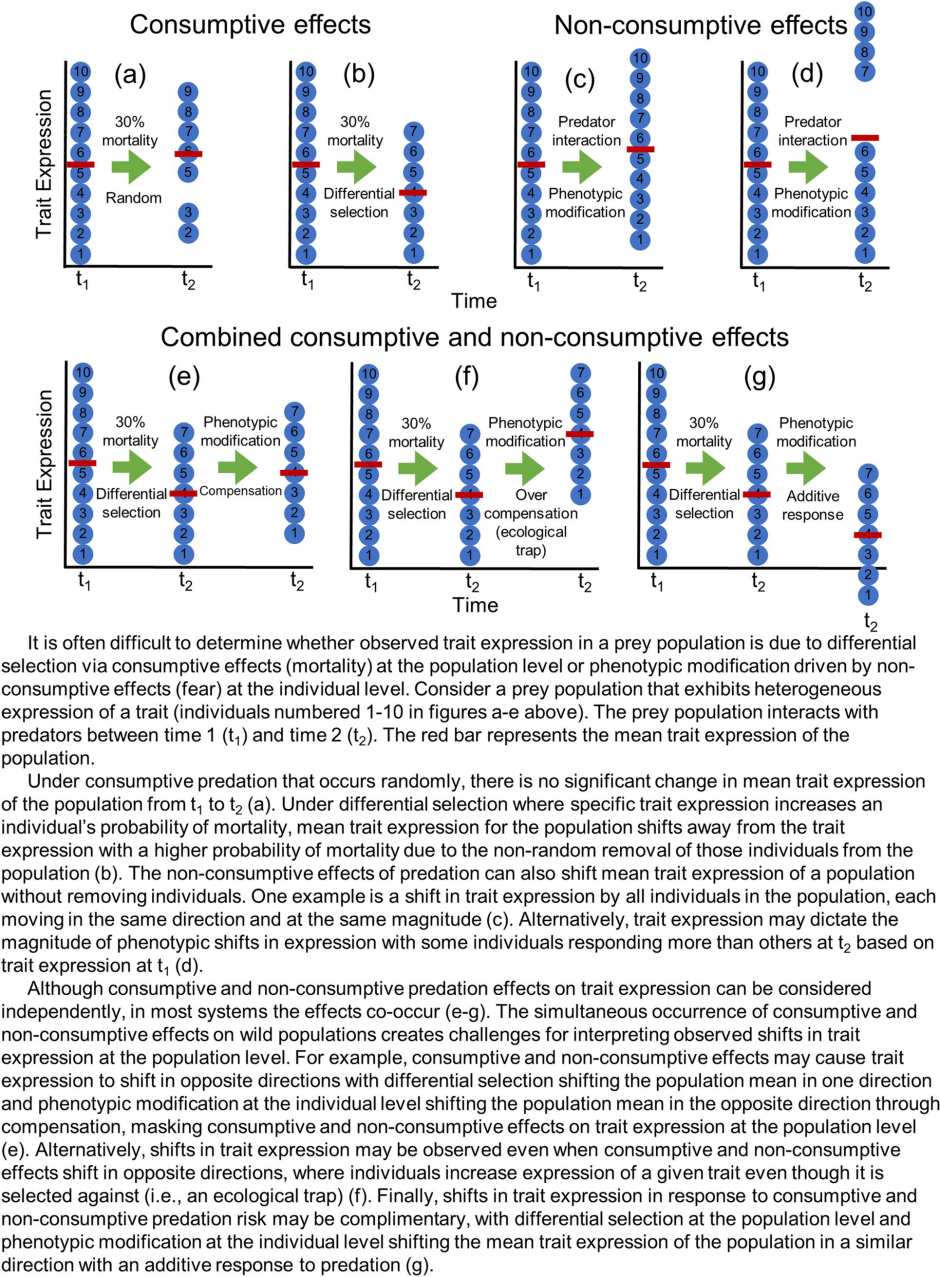
Consumptive and non-consumptive predation effects on trait expression.

Understanding how predators influence where prey are found is further complicated by an individuals’ perception of predation risk. An emerging body of evidence suggests that the perception of predation risk alters space use, time allocation, species distribution, population growth, and species interactions [14,18,26–32]. Moreover, theory, as well as empirical evidence, illustrates the complicated nature of optimizing trade-offs between competing activities (e.g., foraging, predator avoidance, reproduction, etc.)[14,33,34], and the energetic and fitness implications assumed by individuals that ultimately shape populations [14,18,31,35,36]. Unfortunately, despite the potential importance of perceived predation risk in shaping prey behavior, the evolutionary and ecological scale of interactions between predators and prey, and the complicated nature of such interactions, make it difficult to determine to what extent selection via differential mortality at the population level, versus behavioral modification at the individual level shape observed patterns of prey space use [37]. Do we fail to find individuals using high-risk environments because they have been depredated or because they are expressing risk adverse behaviors (Fig 1)?

Separating the effects of depredation from perceived predation risk is difficult in wild populations, as anti-predator responses are under continual natural selection. Most studies that have isolated the effects of perceived predation risk have done so in laboratory settings by experimentally modifying predators so they are unable to consume prey [30,38], or in the wild by presenting prey with predator cues (e.g., chemical, auditory) in the absence of the predators themselves [18,31,39]. Though such investigations provide insight on the relative effects of depredation and perceived predation risk, they neglect important features of predator-prey dynamics such as feedback from predator-experienced conspecifics that may enhance anti-predator responses even in naïve prey [40]. In the wild, prey receive cues about the presence of predators (e.g., chemical, visual, auditory, olafactory) [41,42], but experiments have necessarily focused on a subset of stimuli, leaving uncertainty regarding how prey respond when presented with a full range of cues. Furthering our understanding of the relative importance of perceived predation risk in shaping prey behavior involves isolating the consumptive effects of predation from the non-consumptive effects of perceived predation risk, and measuring prey behavior at multiple ecological scales under conditions that incorporate spatial and temporal variation in predation risk.

To isolate the effects of perceived predation risk in a wild prey population, we assessed space use of wild Ring-necked Pheasants (*Phasianus colchicus*) in response to spatial and temporal variation in recreational hunting activity by humans. Humans elicit similar anti-predator responses as those prompted by natural predators to which pheasants are also exposed [27,43]. Importantly, regulations regarding hunting of wild pheasant populations limit hunters to harvesting (i.e., depredating) males [44]. As males and females cohabitate, female pheasants receive feedback and reinforcement for the expression of anti-predator responses in the presence of humans. While males experience true predation risk, females only experience perceived predation risk from human hunters, allowing us to disentangle the effects of perceived predation risk from those of depredation on population level behavioral trait expression.

Our objective was to evaluate the relative role of perceived predation risk in shaping space use of pheasants at two spatial scales: seasonal home range and nightly resting locations, here-after referred to as roost sites. Using a before-after-control-impact experimental design, we were able to assess the effects of spatial (between high and low risk sites) and temporal (within and between sites before and throughout the hunting season) variation in predation risk on space use. Moreover, we were able to evaluate the effects of perceived predation risk and depredation on the behaviors expressed within our study populations by independently examining the responses of vulnerable (i.e., male on high risk sites) and invulnerable (i.e., all females) populations to spatial and temporal variation in predation risk. We predicted that if perceived predation risk affected space use, then populations exposed to hunters, independent of actual predation risk (i.e., both males and females on high risk sites), would show changes in space use following the onset of the hunting season. Moreover, assuming that the assessment of predation risk and the corresponding behavioral response is continuous, space use should reflect seasonal declines in hunting pressure that typify pheasant hunting [45,46], such that space use of populations on high risk sites would more closely resemble those on low risk sites as the season progressed. We further predicted that depredation could act either independently or interact with how individuals perceive predation risk to affect space use within our study populations. Specifically, if depredation, not perceived predation risk, affects our measure of space use, then only populations experiencing depredation (i.e., males on high risk sites) would demonstrate changes in space use following the onset of the hunting season. However, if depredation within the population acts in concert with individual behavioral responses to the perception of predation risk, then we could see two alternative outcomes. If hunters differentially depredate individuals that fail to respond to predation risk by altering space use, then changes in space use will be more extreme for populations experiencing depredation (i.e., males on high risk sites). If, however, hunters depredate individuals that would have otherwise altered space use, then changes in space use may be less extreme for populations experiencing depredation (i.e., males on high risk sites) if the perception of predation risk is in part shaped by interactions with predators. Although our predictions broadly encompass space use at any scale, it is reasonable to expect that perceived predation risk may shape prey behavior differently at different scales; therefore, considering space use at multiple scales allows us to evaluate a wider range of potential effects [11,47–49].

## Methods

### Study species

The Ring-necked Pheasant is an introduced, ground-dwelling gallinaceous bird inhabiting grassland and agricultural landscapes throughout the central Great Plains of North America. Pheasants range in mass from 500–1300 grams, stand 50–70 cm tall, and are sexually dimorphic, with males notably larger and distinctly colored [50]. Pheasants are hunted in the fall and winter throughout North America. Hunters pursue pheasants on foot, usually accompanied by dogs, and birds are primarily harvested with shotguns as they flush from vegetation, with hunters permitted to harvest males only [44].

### Study system

We conducted our study from September-January of 2012–2014, starting before the 98-day pheasant hunting season, which opens the last weekend in October and ends 31 January [44]. Our study took place on ten sites in Hitchcock and Hayes counties in Southwestern Nebraska, USA that ranged in size from 29 to 155 hectares. All sites were enrolled in the Conservation Reserve Program and were dominated by warm and cool season grasses with interspersed forbaceous vegetation and minimal woody components. Sites were surrounded by a matrix of agricultural lands (predominately dry-land winter wheat and milo, and irrigated corn and soybeans), rangeland pastures, and other grasslands enrolled in the Conservation Reserve Program. Five sites were open to public hunting and received numerous hunter visits, exposing wildlife inhabitants to relatively high predation risk (‘high-risk’), whereas hunting access to the remaining five sites was restricted (‘low-risk’). On all sites, regulations prohibited killing of female pheasants and legal daily shooting hours were 30 minutes before sunrise to sunset [44]. Natural predators of adult pheasants included mammalian predators such as coyote (*Canis latrans*), red fox (*Vupes vulpes*), and bobcat (*Lynx rufus*), and various raptor species including red-tailed hawk (*Buteo jamaicensis*), great-horned owl (*Bubo virginianus*), prairie falcon (*Falco mexicanus*), and Swainson’s hawk (*Buteo swainsoni*). All predators of adult pheasants are highly mobile and widespread, and predator communities and natural predation rates were not expected to differ between sites (e.g., [51]).

### Assessment of predation risk

We used time-lapse photography to document hunting activity at each site and quantify relative predation risk from hunters. We placed cameras (Moultrie model M880, Bushnell Trophy Cam) at fixed, elevated locations in an arrangement and manner that maximized visualization of the site and minimized overlap in the field of view of each camera. The number of cameras at each site (minimum = 2, maximum = 4, mean = 2.8) varied based on the size of the site, suitable mounting locations, and topography. We mounted cameras at a height, and the field of view was such, that individual hunters were visible but not personally identifiable. We programmed cameras to take one still photograph every five minutes during legal hunting hours each day for the duration of the 98-day pheasant hunting season. We visually inspected each photograph for hunter abundance and recorded the date, time, and site. We summarized predation risk by calculating the mean number of hunters per photo, per site, per day. To account for seasonal reductions in hunting pressure [45,46], and the subsequent consequences for how prey perceive and respond to predation risk, we classified each day of the hunting season as either “early” (27 October– 23 November) or “late” (24 November– 15 December). To explain variation in predation risk during the study period, we formulated a mixed effects model (run in Program R; version 3.1.3, R Development Core Team, 2014 using package “lme4” [52]) following a Gaussian error distribution in a Bayesian framework. Our dependent variable was mean hunters per photo, per site, per day. Risk group (low vs. high), and season (early, late), and their interactions were included as fixed effects and study site was included as a random effect. We simulated draws from the joint posterior distributions of the model parameters for all mixed effects models using non-informative priors with the “sim” function (run in Program R using package “arm”[53]). We extracted the mean and 95% credible intervals (CI) [54] for each parameter based on 5000 simulations. We visually inspected residual plots to assess model fit and deemed them satisfactory.

### Pheasant capture

We captured pheasants via nightlighting [55] from 12–28 September 2012 and 10 September—13 October 2013, and fitted each bird with a 22- or 26-gram necklace style VHF radio transmitter equipped with a mortality sensor (Model #A4070, Advanced Telemetry Systems, Inc., Isanti, Minnesota, USA). Radio collars were placed only when collar mass was less than 5% of an individual’s body mass [56] and a necklace style was selected to minimize behavioral or physical impairment as weight fluctuations on the neck is natural because of food storage in the crop. Basic morphometric measures (mass, tarsus length) were taken, and age (hatch year or after hatch year) and sex were determined by visually inspecting feather color and wear, and spur length and color of males. Individuals were marked with a uniquely numbered aluminum leg band for subsequent identification (via nightlighting or hunter harvest) in the event of radio collar loss or malfunction. All birds were processed and immediately released where captured. Methods were reviewed and approved by the Nebraska Game and Parks Commission (Scientific and Educational Permit 408), and the University of Nebraska-Lincoln Institutional Animal Care and Use Committee (Protocol 1060) and Institutional Review Board (20120912892EX).

### Radio triangulation

We recorded movements of individual radio-tagged pheasants 3–7 days per week from 1 October—15 December via radio telemetry using truck-mounted, null-peak antenna systems [57]. To reflect the full range of habitat needs, we located individuals during times assumed to reflect an individual’s unique daily requirements including foraging (1-hr before sunrise to 2-hr after sunrise and 2-hr before sunset to 1-hr after sunset), loafing (2-hr after sunrise to 2-hr before sunset), and roosting (1-hr after sunset to 1-hr before sunrise), with only one location type represented in a single tracking event. To obtain each location, we took a minimum of three bearings within a 20-minute period to minimize biases and error associated with bird movement [56]. Estimated locations as well as associated error ellipses (calculated based on maximum likelihood estimations; [58]) were processed in the field using on-board computers and Location of a Signal (LOAS) software (Ecological Software Solutions, LLC, Hegymagas, Hungary, Version 4.0). Bearing sets with error ellipses >2000 m^2^ (i.e., >25m radius) were discarded or additional bearings were taken until error ellipse size was <2000 m^2^ for daytime locations (foraging and loafing) and <1000 m^2^ for roost locations. Higher precision for roost sites was desired as roost sites were re-located in the field for vegetative assessment.

Any individual emitting a mortality signal during radio triangulation activities was re-located on the ground using hand-held radio location equipment as weather conditions and time allowed. Date of mortality event, and cause of death or radio collar loss were recorded and radio collars were recovered.

### Space use

#### Home range assessment

A home range is the spatial representation of the area an individual occupies while carrying out activities needed to survive and reproduce [47]. Home range formation is fluid, as individuals continuously weigh and assess changes in sources of selection over space and time and immediately adjust space use in response to current conditions. We assessed home ranges using two different methods. First, we used a traditional method [59,60] by defining and comparing home ranges over three distinct periods. Radiolocations recorded before the onset of hunting season (1 October—25 October, hereafter “pre-hunting season”) were separated from radiolocations recorded during the hunting season and serve as the before-impact control within each risk group. Assuming pheasants continually assess and respond to predation risk, it is reasonable to expect pheasant behavior to change as hunting pressure changes. Therefore, we divided radio telemetry locations recorded during the hunting season into “early” and “late” hunting seasons to reflect the traditional temporal decline in hunting pressure [45,46]. Behavioral responses during the early-hunting season represent an immediate reaction to elevated predation risk, whereas evaluating late-hunting season responses allowed us to determine if immediate behavioral responses continue given a reduction in predation risk as the hunting season progresses. We used radiolocations to estimate 95% and 50% home ranges for each individual during each season (pre, early, and late). Home ranges were estimated using fixed kernel utilization distributions [61,62] (run in Program R using package “adehabitatHR” [63]) with smoothing parameter *h*_ref_. Kernel utilization distributions take into consideration the density of re-locations giving a more accurate assessment of the relative importance of various portions of an individual’s home range, and are less sensitive to outliers, sampling regimes, and detection bias compared to traditional estimators such as minimum convex polygon [64,65]. Individuals with fewer than 10 radiolocations during a given season were excluded from analysis for that season to reduce errors in home range estimation associated with small sample sizes (radiolocations per home range: mean = 17.6, min = 10, max = 25).

There is much debate surrounding issues of autocorrelation, sample sizes, and analysis frameworks for kernel utilization distribution estimation [56,66,67], but it is increasingly apparent that approaches that consider and maintain biologically relevant sampling procedures when exploring animal movement [68,69] are superior to traditional statistically driven frameworks [67,68,70]. Recognizing kernel home range estimations are subject to error, especially with small sample sizes as those presented here [71], our goal was not to estimate and report the ‘true’ size of home ranges, but to measure relative changes in home range size or location between risk groups and seasons. It is reasonable to expect that any error associated with home range estimation due to small sample sizes would not differ between risk groups or season. Assuming consistent error, our limited sample sizes make our assessment conservative, as the increase in error within risk group or season makes detecting differences among groups or seasons more difficult.

To explain variation in home range size in response to predation risk, we formulated a mixed effects model for each sex as described for hunting pressure. Our dependent variable was the size of the 95% home range (hectares), which we log transformed to meet the assumptions of a normal distribution. Risk group (low, high), season (pre, early, late), and their interactions, as well as year were included as fixed effects, and study site and individual were included as random effects. We used a similar model structure to explain variation in home range location in response to predation risk, replacing Euclidean distance (meters) between the pre-season 50% home range center and the early and late-season 50% home range centers as the dependent variable, which we log transformed to meet the assumptions of a normal distribution. Season included only two levels (pre-to-early, and pre-to-late).

Although dividing location data into distinct periods is the traditional approach (pre- vs. early- vs. late-hunting season), important information may be lost as animal movements and hunting pressure are inherently continuous variables. Given the variability in hunting pressure from day-to-day, both within and between sites, and the behavioral response of prey, we developed an analysis that allowed us to treat predation risk and home range size as continuous variables. Using a “moving window” approach, we estimated 95% and 50% home ranges from the same location data by constructing individual home range estimates based on a five-location window, meaning each home range was constructed using 11 re-location events (an individual’s location on a given day plus the five previous and five future re-locations). Windows were “moved” one re-location event (typically 1–2 days) to produce a new home range for the individual, always maintaining 11 re-location events. Because not all individuals were relocated on the same day, our moving window approach resulted in home ranges that varied in time span (days) over which the 11 relocation events took place (minimum = 8, maximum = 38, mean = 13.7). To account for variation in the time represented by each home range we included window length in all models involving the moving window approach. We estimated home ranges using fixed kernel utilization distributions as described earlier. For each 95% home range, we determined the study site it intersected and assigned a measure of hunting pressure for the associated date range by calculating the mean hunters per photo per site per day on that site. In cases where home ranges did not intersect any of our study sites (individuals were ‘offsite’), we assigned predation risk as the mean hunters per photo, per site, per day on our low-risk sites for the relevant date range. Assigning predation risk for offsite home ranges based on risk measured at low-risk sites represents a conservative estimate, as most land was not publically accessible for hunting, but hunting was still possible. To explain variation in home range size in response to predation risk, we formulated a mixed effects model similar to those described above. Our dependent variable was home range size (hectares) log transformed to meet the assumptions of a normal distribution. Sex, date (median date of the home range), window length (days between first and last relocation events), and hunting pressure (mean hunters per photo per site per day) were added as independent variables and study site and individual were included as random effects. To explain variation in home range location related to hunting pressure using our continuous, moving window approach, we evaluated the Euclidean distance between successive 50% home range centers. We attributed each distance moved with the hunting pressure of the previous home range, such that the value represented the predation risk an individual was moving from. To explain variation in home range location in response to predation risk using our moving window approach, we formulated a mixed effects model using a similar structure as explained for our moving window home range size model. We replaced the dependent variable with distance moved (meters), which we log transformed to meet the assumptions of a normal distribution. Fixed and random effects remained the same except predation risk represented risk associated with the previous home range.

We simulated draws from the joint posterior distributions of the model parameters for all mixed effects models using non-informative priors with the “sim” function (run in Program R using package “arm” [53]). We extracted the mean and 95% credible intervals (CI) around the mean [54] based on 5000 simulations. We visually inspected residual plots to assess model fit and deemed them satisfactory.

#### Roost site assessment

Roost sites provide protection from predators and harsh weather conditions [72,73], and thus represent an important measure of space use. Like home range formation, where individuals roost each night changes in response to current conditions; however, as each roost site is embedded within an individual’s home range, the scale of the response is inherently different. We used GPS coordinates of estimated roost site locations acquired via radio-telemetry to physically locate roost sites. We approached the roost during daylight hours when unoccupied and systematically searched the area surrounding the estimated coordinates based on the size of the error ellipse (i.e., maximum 17m search radius) until the presence of fecal matter and feathers confirmed a roost location [74]. To assess vegetation characteristics, we established two 5-m radius plots, one centered on the roost site and another at a non-use site located 35m from the roost site in a randomly selected direction. At each plot, we recorded vegetation cover (percentage warm season grass, cool season grass, bare ground, and litter) using ocular estimation as well as litter depth and visual obstruction (following BBIRD grassland sampling protocol, http://umt.edu/bbird/).

We tested for the effects of risk group, sex, and their interaction, and date on vegetation characteristics with a permutational multivariate analysis of variance (PERMANOVA) using distance matrices (adonis) (run in Program R using package “vegan” [75]). To test for systematic differences in vegetation characteristics among study sites and across the sampling season, we developed a single model using the paired non-use data. We then developed two additional models, one using data from roost sites selected prior to the onset of the hunting season, and the second using data from roost sites selected during the hunting season. Because roost sites for individuals were sampled unevenly within seasons with some individuals measured multiple times and others not at all, we sub-sampled the dataset by randomly selecting one roost per season, per individual to meet the independence assumptions for the adonis analysis. We bootstrapped the adonis results from each model 1000 times, incorporating a new random sample of roost/non-use sites for each iteration. We extracted the median F and lower 2.5% and upper 97.5% bounds and the corresponding p-value for each term in the model, and accepted a result as ‘significant’ if the median p-value was less than α = 0.05.

## Results

### Predation risk

**Fig 2:**
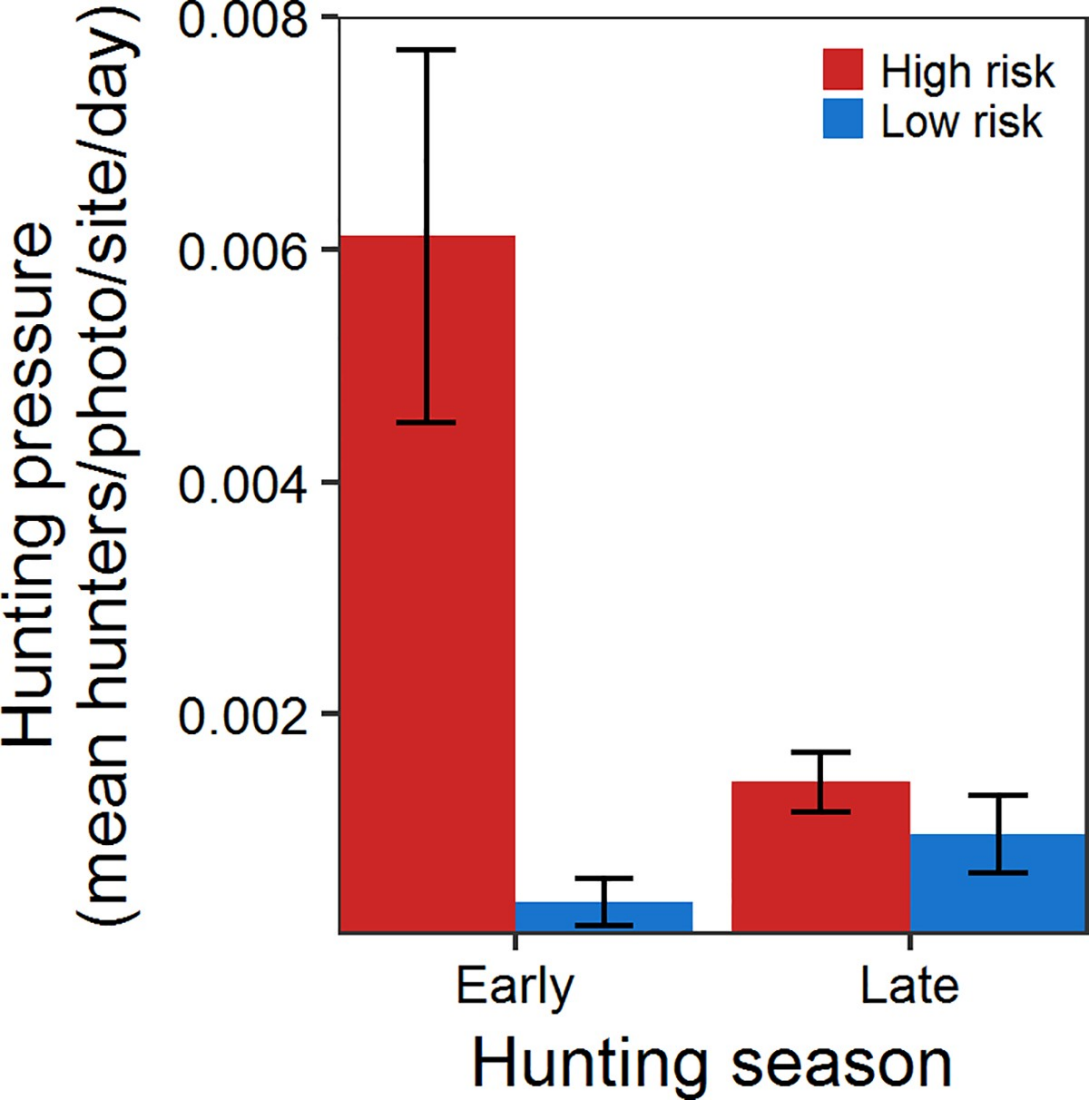
Hunting pressure during the early and late hunting season on high and low risk sites. Hunting pressure (mean number of hunters per photo, per site, per day) on high-risk sites (red bar) early in the hunting season was higher and more variable than hunting pressure on low-risk sites (blue bar) during either season and high-risk sites late in the hunting season. Error bars represent the standard error around the mean.

During the study, 31 cameras across 10 sites took 322,925 images, and detected 590 hunters. Cameras recorded more hunters on high-risk than low-risk sites during the early-season (Fig 2; Tables 1 and 2), but hunter numbers declined in the high-risk group (effect of late season = -0.005, CI: -0.007, -0.003) with predation risk becoming equivocal between groups by the late season (Fig 2; difference between high- and low-risk groups during the late season = 0.00, CI: -0.002, 0.003).

**Table 1:** Descriptive statistics describing hunting pressure and pheasant behaviors. Mean (±S.E.) hunting pressure (hunters per photo per site per day), pheasant home range size (ha) based on 95% kernel utilizations distribution, and shifts in pheasant 50% home range center (m) from the previous season, for two experimental treatments and three seasons with *a priori* expectation of different hunting pressure and thus differences in predation risk.

			Home range size		Home range shift	
Treatment	Season	Hunters	Female	Male	Female	Male
High risk	Pre	–	100.46 (14.29)	168.48 (34.49)	–	–
	Early	0.0061 (0.0016)	442.09 (125.18)	208.77 (40.16)	484.28 (110.71)	363.43 (106.01)
	Late	0.0014 (0.0003)	84.49 (8.94)	133.91 (23.09)	930.08 (278.25)	461.82 (151.16)
Low risk	Pre	–	130.10 (41.33)	165.70 (30.98)	–	–
	Early	0.0004 (0.0002)	108.04 (19.61)	168.25 (32.52)	188.62 (83.50)	269.74 (53.80)
	Late	0.0010 (0.0003)	95.08 (14.28)	119.94 (21.12)	182.35 (40.89)	319.61 (71.77)

**Table 2:** Predictors of predation risk (parameter estimates and 95% credible intervals). Estimates represent differences from the Intercept (high-risk, early-season) and are considered ‘significant’ (bold) if credible intervals do not include zero. Posterior distributions of group-specific means were obtained from the parameterization of each model. If the 95% CI of the difference between the posterior distributions of two means does not overlap zero, we consider the means to be different.

Predictors	*β*^*a*^	q2.5^b^	q97.5^b^
Intercept	**0.006**	**0.005**	**0.008**
Risk group (low)	**-0.006**	**-0.008**	**-0.004**
Season (late)	**-0.005**	**-0.007**	**-0.003**
Risk group (low): Season (late)	**0.005**	**0.002**	**0.009**

^a^ Estimated coefficient (mean of posterior distribution)

^b^ q2.5 and q97.5 = 2.5% and 97.5% quantiles of the posterior distribution

### Space use

We captured and radio-collared 226 pheasants (99 male, 127 female) in 2012 and 2013. Due to constraints on radio triangulation, we included 145 unique individuals (male, low = 35; male, high = 31; female, low = 36; female, high = 43) in the traditional home range size models, and 101 unique individuals (male, low risk = 27; male, high risk = 18; female, low risk = 27; female, high risk = 29) in the traditional home range location models; however, each individual was not necessarily represented during each season. Sample sizes between the home range size and home range location analyses differ due to the paired nature of the home range location analysis. Specifically, distances between pre- and early or late season home ranges could not be calculated for individuals where a pre-, early, or late season home range could not be created when radio-locations during a given season were inadequate (e.g., due to a mortality). Because we treated hunting season and risk as continuous variables in our moving window approach, we were able to include 137 unique individuals (male = 58, female = 79).

We measured vegetative characteristics at 105 roost sites used prior to the hunting season, 188 roost sites used during the hunting season, and 287 non-use sites across all seasons. Due to our random sub-sampling design (to meet independence assumptions of our analysis method), our roost site vegetation analysis included characteristics from 88 roosts used prior to the hunting season (male, low risk = 19; male, high risk = 18; female, low risk = 21; female, high risk = 33), and 133 roosts used during the hunting season (male, low risk = 30; male, high risk = 19; female, low risk = 39; female, high risk = 45), which we paired with 218 non-use locations (male, low risk = 48; male, high risk = 36; female, low risk = 60; female, high risk = 74).

### Home range

**Fig 3:**
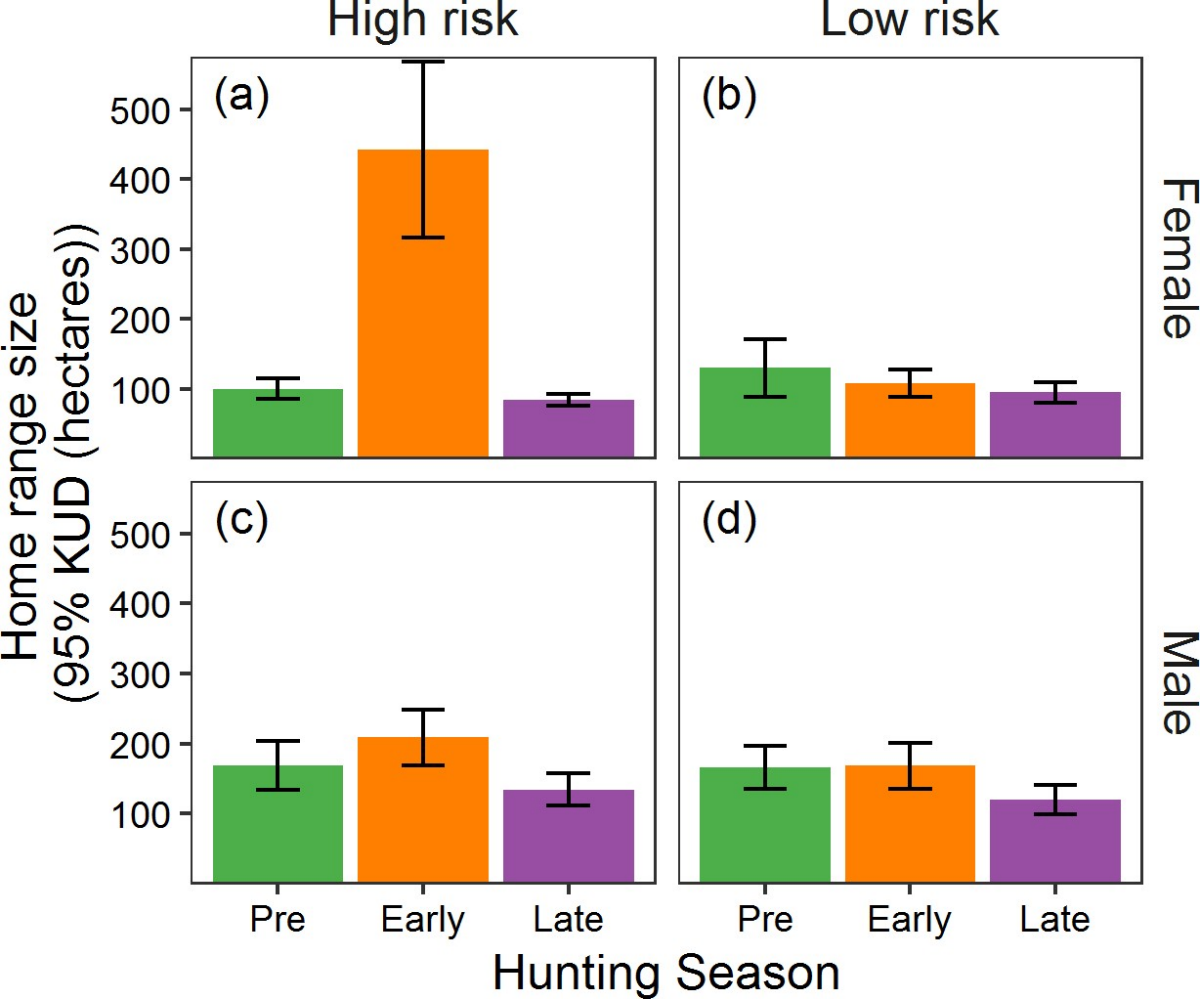
Our traditional approach found that only female pheasants in the high-risk group showed an increase in home range size in response to the onset of the hunting season (Table 2). Colored bars represent mean home range size (hectares) for female and male pheasants in high- and low-risk groups before (pre) and during (early and late) the hunting season. Error bars represent the standard error around the mean.

**Fig 4:**
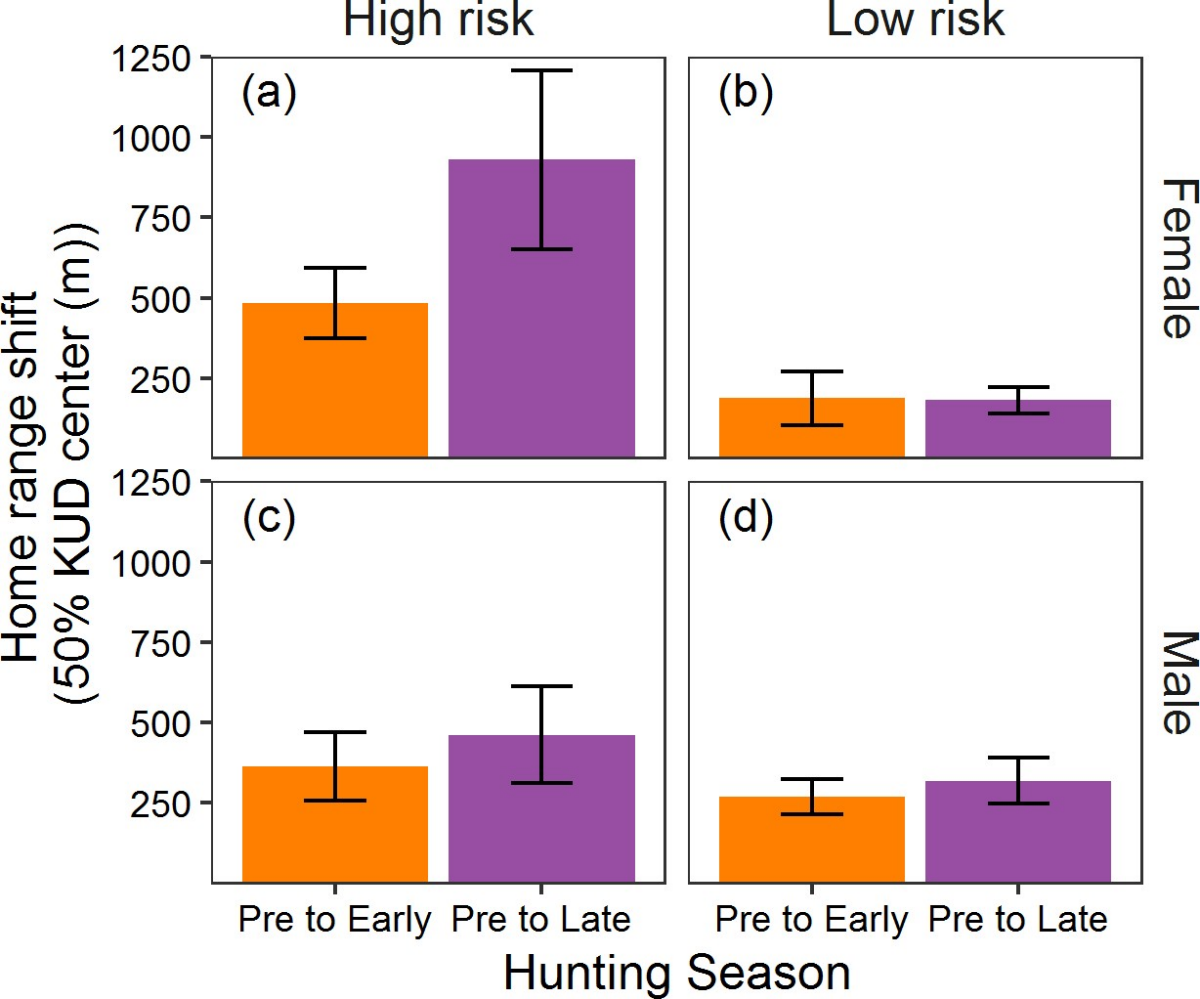
Our traditional approach revealed a shift in home range center for all pheasants, but only female pheasants occupying high-risk sites shifted home range center in response to the onset of the hunting season. Colored bars represent mean shift home range center for males and females in each risk group from the pre- to early hunting season (orange bar) and from the pre- to late hunting season (purple bar). Errors bars represent the standard error around the mean.

The traditional home range analysis revealed that female pheasants on high-risk sites increased home range size during the early pheasant-hunting season (Fig 3A and 3B; Table 3), but the effect decreased by the late hunting season when home range sizes were not different from the pre-season. There was no significant effect of risk group or season on male pheasant home range size (Fig 3C and 3D; Tables 1 and 3). For female pheasants on high-risk sites, the traditional analysis revealed a significant shift in home range center (Fig 4A and 4B; Tables 1 and 3), with the effect apparent throughout the hunting season. There was no effect of predation risk on male home range location; however, males on all sites shifted home range center as the hunting season progressed (Fig 4C and 4D; Tables 1 and 3).

**Table 3:** Predictors of home range size and location (parameter estimates and 95% credible intervals)—traditional analysis. Estimates represent differences from the Intercept (low-risk, pre-season, 2012) and are considered ‘significant’ (bold) if credible intervals do not include zero. Posterior distributions of group-specific means were obtained from the parameterization of each model. If the 95% CI of the difference between the posterior distributions of two means does not overlap zero, we consider the means to be different.

			Female				Male		
Model	Predictors		*β*^*a*^	q2.5^b^	q97.5^b^		*β*^*a*^	q2.5^b^	q97.5^b^
Size	Intercept		**4.83**	**4.40**	**5.25**		**5.21**	**4.73**	**5.70**
	Risk group (high)		-0.03	-0.56	0.50		0.01	-0.60	0.63
	Season (early)		0.09	-0.29	0.49		0.17	-0.15	0.49
	Season (late)		0.09	-0.34	0.51		-0.09	-0.46	0.29
	Year (2013)		**-0.68**	**-0.98**	**-0.39**		**-1.01**	**-1.36**	**-0.67**
	Risk group (high): Season (early)		**0.77**	**0.25**	**1.31**		0.14	-0.36	0.65
	Risk group (high): Season (late)		-0.21	-0.77	0.36		0.03	-0.51	0.57
Location	Intercept		**5.21**	**4.73**	**5.70**		**5.57**	**4.80**	**6.34**
	Risk group (high)		0.01	-0.60	0.63		0.12	-0.87	1.09
	Season (pre to late)		0.17	-0.15	0.49		**0.42**	**0.10**	**0.75**
	Year (2013)		-0.09	-0.46	0.29		**-0.75**	**-1.29**	**-0.21**
	Risk group (high): Season (pre to late)		**-1.01**	**-1.36**	**-0.67**		-0.15	-0.62	0.33

^a^ Estimated coefficient (mean of posterior distribution)

^b^ q2.5 and q97.5 = 2.5% and 97.5% quantiles of the posterior distribution

**Fig 5:**
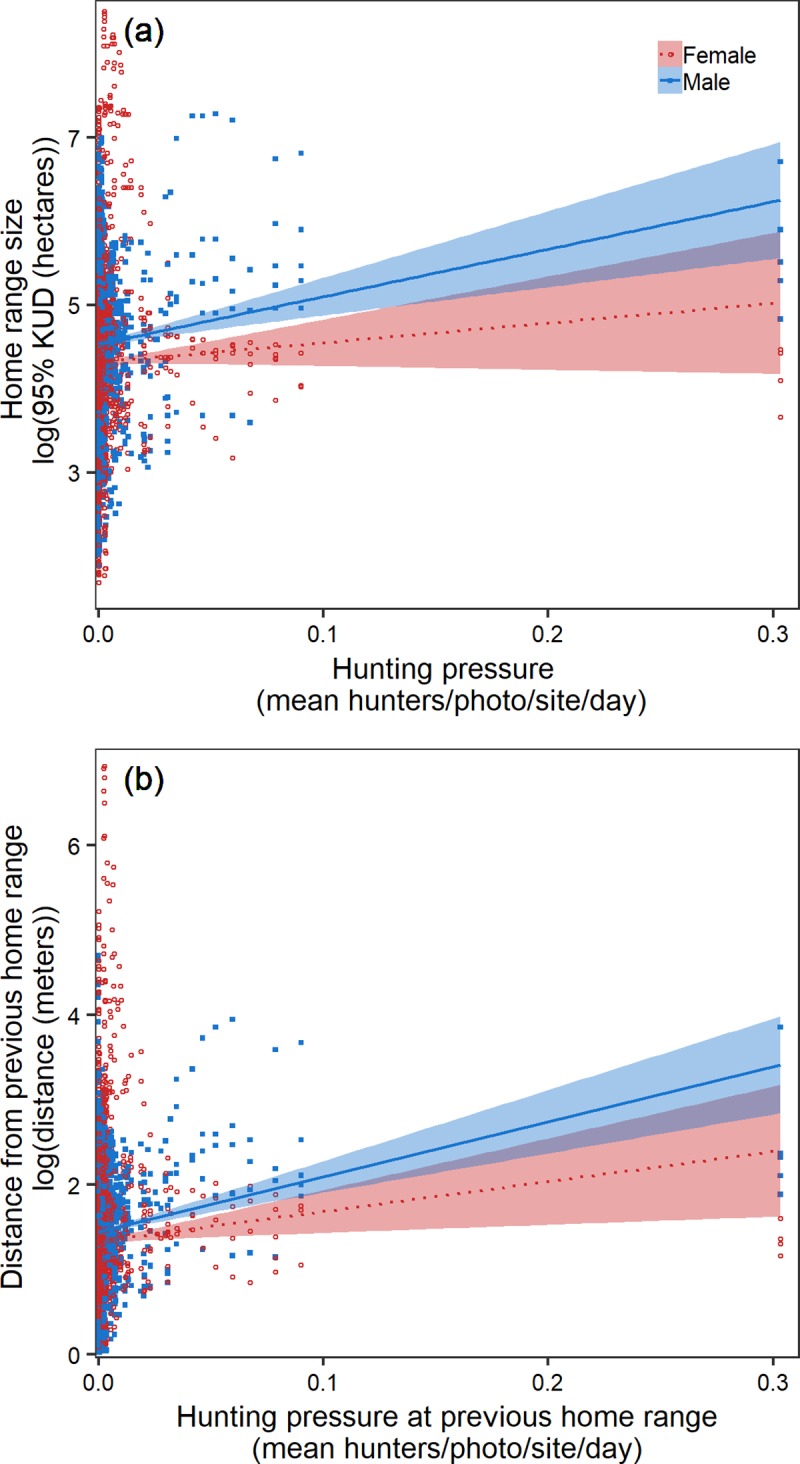
**When hunting pressure and time were considered continuously rather than as discrete groups, there was a positive relationship for males and females between home range size and predation risk (a) as well as the distance moved between consecutive home ranges and predation risk (b).** Linear regression of the relationship between home range size and distance moved for males (solid blue line) and females (dotted red line) with shaded error ribbons representing the standard error around the mean.

Our moving window analysis revealed that the home range size of male and female pheasants increased by an average of 9.39 hectares with every one hunter per photo, per site, per day increase in hunting pressure (Fig 5A; Table 4). Shifts in home range center were also positively related to hunting pressure, as individuals shifted home range location 3.82 meters when their previous home range location experienced one hunter per photo, per site, per day (Fig 5B; Table 4).

**Table 4:** Predictors of home range size and location (parameter estimates and 95% credible intervals)—moving window analysis. Estimates represent differences from the Intercept (female, 2012) and are considered ‘significant’ (bold) if credible intervals do not include zero. Posterior distributions of group-specific means were obtained from the parameterization of each model. If the 95% CI of the difference between the posterior distributions of two means does not overlap zero, we consider the means to be different.

	Size			Location		
Predictors	*β*^*a*^	q2.5^b^	q97.5^b^	*β*^*a*^	q2.5^b^	q97.5^b^
Intercept	**4.70**	**4.39**	**5.02**	**1.69**	**1.30**	**2.08**
Log mean hunters	**2.24**	**0.83**	**3.72**	**1.34**	**0.43**	**2.26**
Sex (male)	0.18	-0.09	0.45	0.07	-0.14	0.28
Median season day	**-0.005**	**-0.006**	**-0.003**	**-0.003**	**-0.004**	**-0.002**
Window length	**0.05**	**0.04**	**0.06**	**0.03**	**0.03**	**0.04**
Year (2013)	**-1.28**	**-1.42**	**-1.13**	**-0.86**	**-0.97**	**-0.76**

^a^ Estimated coefficient (mean of posterior distribution)

^b^ q2.5 and q97.5 = 2.5% and 97.5% quantiles of the posterior distribution

### Roost site

We saw consistent patterns in all three of our model sets as our pre-season, within season, and non-use vegetation data all showed significant differences between risk groups and across the sampling period, but not between sexes (Table 5).

**Table 5:** Predictors of pre- and within season and non-use roost site vegetative characteristics (median F statistic, p-values and respective 95% credible intervals). Results were accepted as ‘significant’ (bold) if the median p-value was less than α = 0.05.

Model	Predictors	*F*^*b*^	p^c^
Pre _1,87_^a^	Risk group	**4.87 (3.82, 6.00)**	**0.004 (0.011, 0.001)**
	Sex	0.62 (0.27, 1.10)	0.587 (0.844, 0.340)
	Risk group: Sex	0.630 (0.15, 1.32)	0.623 (0.917, 0.256)
	Date	1.96 (0.98, 3.48)	0.110 (0.397, 0.012)
Within _1,132_ ^a^	Risk group	**3.63 (2.29, 5.40)**	**0.010 (0.066, 0.002)**
	Sex	1.26 (0.61, 2.18)	0.29 (0.662, 0.081)
	Risk group: Sex	0.80 (0.35, 1.71)	0.510 (0.820, 0.153)
	Date	**2.91 (1.38, 5.13)**	**0.023 (0.258, 0.002)**
Non-use _1,217_ ^a^	Risk group	**6.58 (5.36, 7.94)**	**0.001 (0.005, 0.001)**
	Sex	0.14 (-0.16, 0.51)	0.935 (0.994, 0.692)
	Risk group: Sex	0.23 (-0.06, 0.59)	0.881 (0.990, 0.646)
	Date	**16.86 (14.13, 19.56)**	**0.001 (0.001, 0.001)**

^a^ Model and corresponding degrees of freedom

^b^ Median F statistic (lower 2.5%, upper 97.5%)

^c^ Median p-value (lower 2.5%, upper 97.5%)

### Mortality

**Fig 6:**
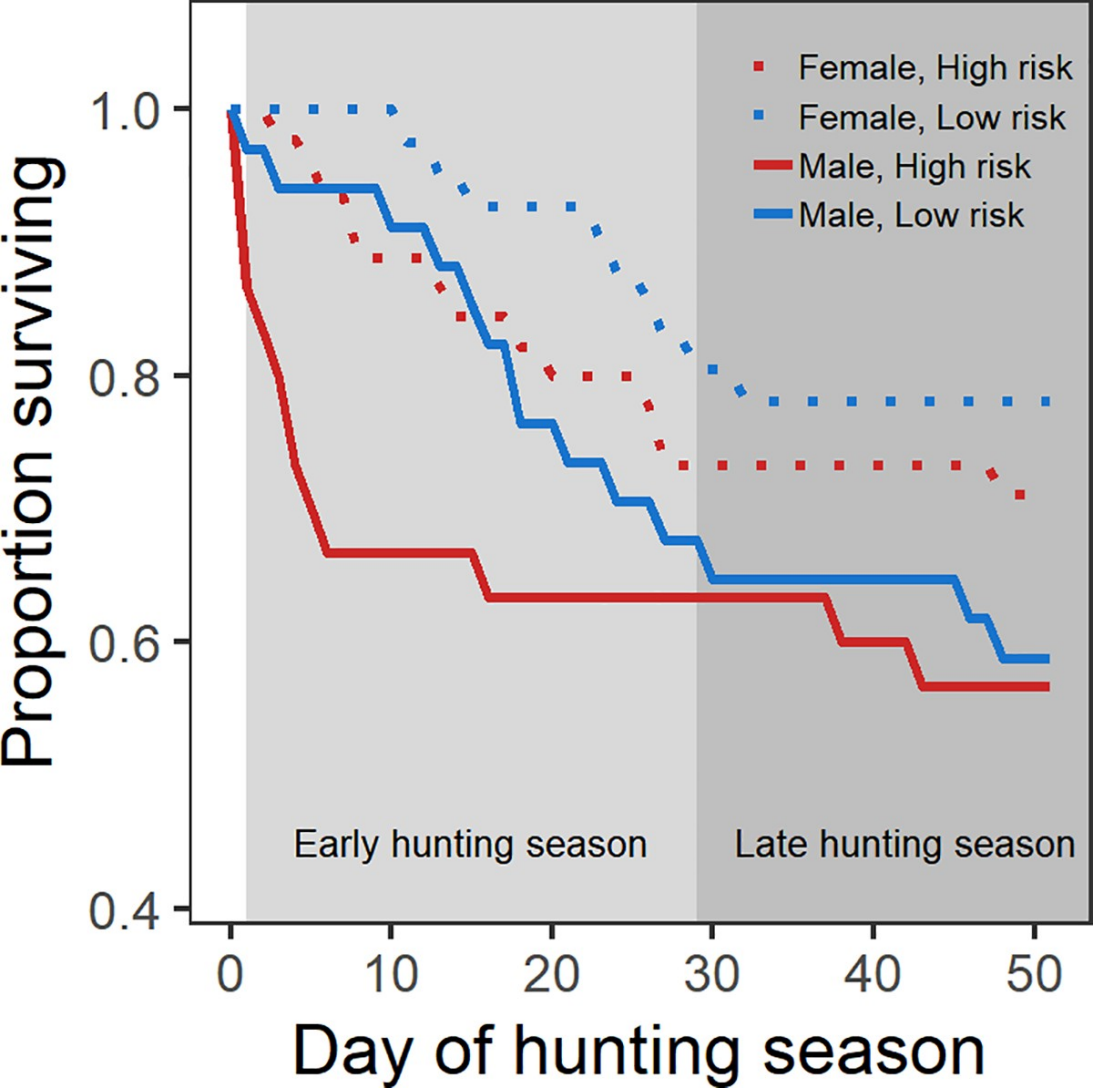
Males on high risk sites experienced higher mortality at the onset of hunting season, but by the end of the early hunting season, the proportion of the initial populations of males surviving on high and low risk sites were similar. Proportion of male (solid lines) and female (dotted lines) pheasants surviving during the early (light gray shaded region) and late (dark gray shaded region) hunting seasons on high (red) and low (blue) risk sites.

Of the 150 radio collared pheasants alive at the onset of the hunting season, we recorded 49 total mortality events during the hunting season (Fig 6). The mortality for males on high risk sites was 30% during the first 10 days of the hunting season, with a majority of mortality events occurring in the first 5 days. Comparatively, males on low risk sites experienced 9% mortality in the first 10 days of the hunting season. Females on high risk sites experienced 11% mortality in the first 10 days of the hunting season, with no recorded mortality events for females on low risk sites during the same timeframe.

## Discussion

We simultaneously assessed the response of a vulnerable (male) and invulnerable (female) prey population to spatial and temporal variation in predation risk, and show that predation risk alone can alter space use, but that depredation within the prey populations can obscure such effects if the responses are not measured at the appropriate spatial and temporal scale. Indeed, we show that the response of the prey population is scale specific and likely reflects the spatial and temporal scale with which prey interact with predators and assess trade-offs with other important ecological processes that affect space use. Over three periods with distinct differences in predation risk, the population exposed to only the perception of predation risk increased home range size and shifted home range centers between the pre- and early-hunting seasons on risk-high risk, but not low risk, sites (Figs 3 and 4). Moreover, the invulnerable population proved sensitive to temporal variation in predation risk, as changes in home range formation were reversed on high-risk sites once risk subsided (Fig 3). The dramatic response to the perception of predation risk suggests that home range formation is highly plastic in response to spatial and temporal variation in predation risk, and begs the question of why we failed to see a similar response in the vulnerable population when using the same assessment (Figs 3 and 4).

One reason may be the true challenge of separating the inherent interaction between behavioral modification at the individual level and differential mortality at the population level [76–78]. We observed extreme variation in early-season home range formation among members of the invulnerable population (Fig 3A), but not the vulnerable population (Fig 3C) that may highlight how differential mortality affects behavioral expression measured at the population level. The average home range of females on high-risk sites doubled from the pre- to early-hunting seasons, but individual responses ranged from a 1052% decrease to a 98% increase. Perceived predation risk manifests individually [79,80], and responses are likely a complex process unique to each individual as trade-offs inherent in home range adjustments are likely sensitive to a range of factors including sex, age, experiences, body condition, resource availability, familiarity with the surrounding landscape [81–84], and maybe most importantly, predator encounter rate [21]. Although there were substantial differences in the number of hunters between high- and low-risk sites (Fig 2), hunter movements within sites ultimately determine encounter rates for individual pheasants [85]. Females that did not alter home range formation may have simply occupied safer locations (i.e., lower predator encounter rates) within high-risk sites. Assuming the same pattern holds true, males on high-risk sites that survived the onset of the hunting season may have simply had fewer encounters with hunters, and thus no need to alter their home range. In contrast, males that had multiple encounters with hunters were at greater risk of being harvested and were removed from the population before expressing a measurable response (i.e., adjusting their home range). Thus it is possible that differential mortality between prey that encounter predators, and those that don’t, may mask the potential effects of perceived predation risk on a population (Fig 1E).

The inconsistency we documented in home range formation between our vulnerable and invulnerable populations also highlights the importance of measuring outcomes at temporal scales that reflect the ecological processes affecting predator-prey interactions [11,23,86]. The complexities of measuring a temporally dynamic response are clearly compounded when the process being measured, predation, inherently affects your sampling effort. For example, given that only 15–30% of encounters between hunters and vulnerable pheasants (i.e., males) result in mortality [87,88], we would assume that vulnerable individuals are afforded an opportunity to alter behaviors in response to changing predation risk on the landscape. Still, even with low depredation rates, 85% of the total hunter-related mortality occurred during the early hunting season (Fig 6). As many males died before we recorded sufficient locations in the early-season, our sample size of males was reduced, resulting in our limited ability to detect a change in home range formation. However, using an approach with a resolution that more accurately reflects the temporal scale of predator-prey interactions, we show that independent of true vulnerability, pheasants increase home range size in response to predation risk on the landscape (Fig 5). Increasing the temporal resolution in our behavioral assessment allowed us to describe an individuals’ home range during the most important period shaping home range formulation, and thus facilitated a more detailed understanding of predator-prey interactions that reflects the temporal scale that drives behavioral trait expression within the population. Clearly, the dynamic nature of risk assessment allows individuals to continually adjust behaviors to reflect changes in predation risk [89,90], as is demonstrated by the response of our invulnerable population to the seasonal decline in predation. Our capacity to detect such changes is ultimately subject to our ability to measure responses at appropriate scales.

The importance of scale in shaping and measuring responses to predation risk is further illustrated when we consider roost sites. Unlike shifts in home range formation, the use of roost sites appears insensitive to predation risk. Although there were differences among risk groups, the differences reflect inherent differences among study sites, not responses to predation risk. That we failed to find a relationship between predation risk and roost site use is initially surprising, but when we considered the temporal scale of predator-prey interactions and the subsequent response of the prey, our findings appear less unexpected. Pheasants typically select, and subsequently occupy, roosts after hunting is closed for the day. If the assessment of risk is timed with the selection process [14], we might not expect roost site use to be responsive simply because there is insufficient information to indicate a change is necessary. Moreover, based on the significant variation in how predation risk shapes home range formulation, it appears that the response of pheasants to predation risk may reflect predator-prey encounter rates, and not the presence of predators on the landscape per se. Pheasants may simply not encounter hunters when occupying roosts, thus limiting the feedback necessary to initiate a shift in roost site use.

Even if pheasants do encounter hunters at the roost, the hierarchical nature of space use [91,92] and increasing evidence that ecological processes manifest at specific spatial and temporal scales [93,94], suggests that behavioral responses at the spatial scale of a roost site may not be necessary [92]. When exposed to high predation risk, pheasants clearly alter home range formation, presumably shifting home ranges to locations with lower predation risk. Upon occupying a location with lower risk, it is presumably not necessary to then alter roost sites to be ‘safer’, as there is no additional benefit. Ironically, the relative safety of the roost site, not the home range, may be the primary determinant of the source of feedback initiating changes in space use, but the safety of a home range, not the roost site, appears to guide subsequent space use. Empirical studies of wildlife-habitat relationships abound [7,8,10,12,95] including examinations of the effects of predation risk on space use [13,15–17,60]; however, despite evidence that wildlife-habitat relationships must be measured at the appropriate ecological scale [93] few studies have considered the consequences of predation risk across multiple spatial scales (but see [96,97]. Although our results are not definitive, they suggest that trade-offs among sources of natural selection, and in particular predation risk, likely act across ecological scales to ultimately shape space use within a population.

Interactions with predators clearly have implications for prey and in some instances entire ecosystems [1–3,5]. The extent to which the direct effects of predators on prey populations (i.e., predation rate), versus the indirect effects of predators on prey individuals (i.e., perceived predation risk) drive trait expression in wild populations remains largely unresolved [98]. For example, there is ongoing debate over the relative importance of direct and indirect predator effects on trophic cascades in natural systems [37,99–101]. Our data cannot address such a complex controversy; yet we would caution, as others have (e.g., [102]), that it is challenging to rely on correlates of predation risk for assessing the role of perceived predation risk in shaping ecological systems. Predation risk and predation rate are not mutually exclusive, and can shape systems in very different ways (Fig 1). Indeed, without our ability to independently assess the indirect and direct effects of hunting on pheasant space use, we may have come to very different conclusions. We also argue that representing how prey perceive risk requires scale-appropriate assessments of risk measured with an appropriate level of resolution. Ecological conditions are increasingly identified as scale-dependent [11,47–49,51,103] and even in our simple system slight differences in the scale (weeks versus months, home range versus roost site) at which we considered risk and measured the response of prey had significant implications. Predation risk, predation rate, and the corresponding scale of response are highly species and context dependent [47,104], which may ultimately limit our understanding of how perceived predation risk shapes ecological systems. Given the complexities of spatial and temporal variation in predation risk and predation rate [15,21,105] we argue that understanding wild prey populations requires tests that simultaneously and independently alter risk and mortality while measuring outcomes at ecologically appropriate scales.
